# Prevalence of rheumatoid arthritis in China: Variations and trends from the global burden of disease study 2021

**DOI:** 10.1515/rir-2025-0013

**Published:** 2025-07-01

**Authors:** Yan Cheng, Tihong Shao, Xingdong Chen

**Affiliations:** State Key Laboratory of Genetic Engineering, Human Phenome Institute, and School of Life Sciences, Fudan University, Shanghai 201203, China; Department of Rheumatology and Immunology, The First Affiliated Hospital of Anhui Medical University, NO 228 Jixi Road, Hefei 230022, Anhui Province, China

**Keywords:** rheumatoid arthritis, prevalence, incidence, disability-adjusted life years, GBD 2021

## Abstract

**Background and Objectives:**

The latest prevalence data for rheumatoid arthritis (RA) is essential for effective disease control and management. Although numerous studies examine the global burden of RA, recent data specific to China has been lacking. The aim of this study is to evaluate the variations of RA burden in mainland China from 1990 to 2021 utilizing data from the Global Burden of Diseases, Injuries, and Risk Factors (GBD) study 2021.

**Methods:**

We conducted a retrospective analysis of RA burden in China based on the GBD 2021 study. We collected data on RA prevalence, incidence, and disability adjusted life years in China from 1990 to 2021, then calculated the estimated annual percentage change (EAPC) to summarize the overall trends.

**Results:**

In 2021, the total numbers of prevalent cases, incident cases, and disability-adjusted life years (DALYs) for RA in China were 4,755,500, 247,300, and 833,800, respectively; From 1990 to 2021, the rates of incidence, prevalence, and DALYs of RA showed an increasing trend, with EAPCs of 0.61, 0.59, and 0.17, respectively. Throughout this period, the RA burden was notably higher in females than in males. In 2021, RA incidence rates among individuals aged 15–44, 45–59, and 60–85+ followed an approximate 1∶1∶1 ratio. Projections using the Bayesian Age-Period-Cohort (BAPC) model indicated that by 2045, compared to 2021, the total incident cases, prevalent cases, and DALYs of RA in China are expected to increase by 15.7%, 13.2%, and 4.2%, respectively.

**Conclusion:**

Over the past 32 years, the RA burden in China had continued to increase, and is expected to rise substantially over the next decade. Therefore, it is necessary to strengthen the prevention and management of RA, with priority given to the female population and young patients.

## Introduction

Rheumatoid arthritis (RA) is a chronic autoimmune disease characterized by systemic autoimmune disorders and synovial inflammatory, affecting an estimated 0.2% to 1.0% of the global population.^[1]^ Evidence indicates that RA patients require ongoing medical care and pharmacologic intervention, which not only drives up healthcare costs but also contributes to significant work disability and reduced quality of life, thereby imposing a substantial economic burden on individuals and society.

Assessing the latest prevalence of RA at both global and national levels is crucial for effective disease control and management. Numerous studies in recent years have examined RA prevalence worldwide.^[2, 3, 4, 5, 6, 7, 8]^ The latest findings by the Global Burden of Diseases, Injuries, and Risk Factors (GBD) 2021 Rheumatoid Arthritis Collaborators revealed that: in 2020, an estimated 17.6 million (95% uncertainty interval 15.8–20.3) individuals were affected by RA globally, with an age-standardized prevalence rate of 208.8 cases per 100, 000 population (186.8–241.1) and an age-standardized disability-adjusted life year (DALY) rate of 36.4 per 100, 000 population (27.6–45.9).^[9]^ Given China’s large population, recent research specific to RA burden has been limited. Existing studies primarily focus on satisfaction with drug treatments among RA patients from a quality-of-care perspective,^[10]^ or on the economic implications of RA,^[11]^ with an absence of recent epidemiological data.

This study leverages GBD 2021 data to analyze trends in RA prevalence in mainland China from 1990 to 2021; and applies the Bayesian Age-Period-Cohort (BAPC) model to predict the trend of RA prevalence from 2022 to 2045. The findings contribute to the literature by offering critical insights for healthcare policy, resource allocation, and the design of prevention and intervention strategies to mitigate the RA burden in China.

## Methods

### Data Resources

This study utilized data from the Global Burden of Diseases, Injuries, and Risk Factors Study 2021,^[9]^ which provides comprehensive metrics on prevalence, incidence, mortality, years lived with disability (YLDs), years of life lost (YLL), and DALYs of 369 diseases and injuries in different sex and age groups within 204 countries and territories, 7 super-regions, and 21 regions from 1990 to 2021. GBD, published by the Institute for Health Metrics and Evaluation (IHME), represents one of the most exhaustive studies of global diseases, injuries, and risk factors. Data sources for GBD include systematic reviews of published studies, governmental and international databases, published reports, and primary datasets, such as the Demographic and Health Surveys. Standardized steps in GBD data processing include input data standardization, age-sex stratification, cause aggregation, and noise reduction, followed by analysis through major models such as the cause of death ensemble model, spatiotemporal Gaussian process regression, and DisMod-MR.^[12]^ The data were retrieved from the Global Burden of Disease Data Exchange Results Tool (https://vizhub.healthdata.org/gbd-results/).^[13]^ This study selected “Rheumatoid arthritis” as the cause of interest, and “prevalence”, “incidence”, and “DALYs ” as measures. DALYs, an indicator of disease burden, combine years of life lost to premature mortality with years lived with disability.

### Statistical Analysis

#### Calculation of ASPR, ASIR and ASDR

In addition to basic indicators such as the numbers of prevalent cases, incident cases, and DALYs, this study also employed age-standardized prevalence rate (ASPR), age-standardized incidence rate (ASIR), and age-standardized DALYs rate (ASDR) to assess the burden of RA. The formula for calculating the age-standardized rate (ASR) is as follows:


$$ASR=\frac{\sum_{i=1}^{A}a_{i}w_{i}}{\sum_{i=1}^{A}w_{i}}\times 100,000$$

where *i* represents the *i*th age group, *ai* represents the specific disease crude rate of the *i*th age group, and *wi* represents the reference standard for selection, and weight of the *i*th age group in the population.^[14]^

#### Trend Analysis

The estimated annual percentage change (EAPC) was used to quantify changes in ASR over time. The 95% confidence interval (CI) for the EAPC was derived using a linear regression model, with EAPC values indicating the annual percent change (increase, decrease, or stability). For instance, an EAPC of 0.1 denotes a 0.1% annual increase.


$$\begin{array}{r} In(ASR)=\alpha +\beta (calendaryear)+\varepsilon \\ \;EAPC=100\times (exp(\beta )-1)\\ 95\text{\%}CI=\beta \pm 1.96S\beta (S\beta =S/\sqrt{n}) \end{array}$$

where α in the formula represents the regression intercept, β indicates the slope of the regression line, representing the trend over time, ε represents random error and *n* is the number of independent variables.^[14]^

Projections of RA prevalence from 2022 to 2045 were made using the BAP) model, which accounts for age, period, and cohort effects to forecast incidence, prevalence, and DALYs. Model performance was validated by comparing projected values to observed data from 2010 to 2021. All statistical analyses were conducted using R software (version 4.2.1), with significance set at *P* < 0.001.

## Results

### National Overall Trends

The incidence rate of RA in China rose from 11.6 per 100,000 population (95% CI: 10.1–13.2) in 1990 to 13.7 per 100,000 population (95% CI: 12.1–15.6) in 2021, reflecting a statistically significant increase (EAPC = 0.61, 95% CI: 0.58–0.64, *P* < 0.001). Similarly, the prevalence rate of RA increased from 205.7 per 100, 000 population (95% CI: 177.6–238.2) in 1990 to 240.7 per 100,000 population (95% CI: 210.8–277.9) in 2021, with an EAPC of 0.59 (95% CI: 0.55–0.63, *P* < 0.001) (Table 1). The DALYs rate exhibited a moderate increase, rising from 42.2 per 100,000 population (95% CI: 31.3–55.4) in 1990 to 42.4 per 100,000 population (95% CI: 33.0–54.3) in 2021, with an EAPC of 0.17 (95% CI: 0.05–0.29, *P* = 0.008) (Table 1). These trends indicate a growing burden of RA in China over the past 32 years.

**Table 1:** Number and age-standardized rate of RA prevalence, incidence, and DALYs in China in 1990 and 2021 for male, female, and both sexes.

	1990		2021		EAPC in rate, 1990-2021	*P* value
	Cases (×103)	Rate	Cases (×103)	Rate		
Incidence (95% CI)						
Both	127.8 (111.5, 145.9)	11.6 (10.1, 13.2)	247.3 (216.2, 283)	13.7 (12.1, 15.6)	0.61 (0.58, 0.64)	<0.001
Male	42.3 (36.6, 49.1)	7.9 (6.9, 9.1)	90.6 (79.2, 104)	9.9 (8.7, 11.2)	0.73 (0.71, 0.76)	<0.001
Female	85.5 (74.2, 97.4)	15.6 (13.6, 17.7)	156.7 (137.3, 179.8)	17.8 (15.7, 20.2)	0.52 (0.48, 0.56)	<0.001
Prevalence (95% CI)						
Both	2041.7 (1746.9, 2391.0)	205.7 (177.6, 238.2)	4755.5 (4141.2, 5452.5)	240.7 (210.8, 277.9)	0.59 (0.55, 0.63)	<0.001
Male	628.6 (530.9, 752.4)	128 (109.8, 150.1)	1558.8 (1345.2, 1823.4)	160.8 (139.4, 187.5)	0.76 (0.73, 0.79)	<0.001
Female	1413. 1 (1210.8, 1646.8)	286.2 (247.9, 332.5)	3196.7 (2798.2, 3652.4)	321.7 (280.0, 369.7)	0.47 (0.42, 0.52)	<0.001
DALYs (95% CI)						
Both	403. 1 (307.6, 526.2)	42.2 (31.3, 55.4)	833.8 (621.5, 1083.5)	42.4 (33.0, 54.3)	0. 17 (0.05, 0.29)	0.008
Male	125.8 (92.6, 166.9)	27.6 (20.4, 36.3)	290.3 (215, 382.3)	30.6 (22.7, 40.3)	0.55 (0.39, 0.71)	<0.001
Female	277.2 (209.7, 360.6)	57.6 (44.3, 74.0)	543.5 (400.1, 721.5)	54.1 (39.3, 72.6)	-0.04 (-0.15, 0.07)	0.442

RA: rheumatoid arthritis, DALYs: disability-adjusted life years, EAPC: estimated annual percentage change, CI: confidence interval.

By 2021, the total number of prevalent RA cases in China had increased to 4,755,500 cases (95% CI: 4,141,200–5,452,500), representing a 2.3-fold rise from 1990 levels of 2, 041, 700 cases (95% CI: 1,746,900–2,391,000)(Table 1). Similarly, incident cases increased 1.9-fold from 127, 800 cases (95% CI: 111,500–145,900) in 1990 to 247, 300 cases (95% CI: 216,200–283,000) in 2021, while the total number of DALYs rose 2.1-fold from 403, 100 cases (95% CI: 307,600–526,200) in 1990 to 833, 800 cases (95% CI: 621,500–1,083,500) in 2021 (Table 1).

### National Trends by Sex

From 1990 to 2021, the incidence rates of RA increased in both males and females. Among males, the incidence rate rose from 7.9 per 100, 000 population (95% CI: 6.9–9.1) in 1990 to 9.9 per 100,000 population (95% CI: 8.7–11.2) in 2021, with an EAPC of 0.73 (95% CI: 0.71–0.76, *P* < 0.001). While in females, the incidence rate increased from 15.6 per 100,000 population (95% CI: 13.6–17.7) to 17.8 per 100,000 population (95% CI: 15.7–20.2), with an EAPC of 0.52 (95% CI: 0.48–0.56, *P* < 0.001) (Table 1). Similarly, RA prevalence rates rose significantly over this period in both sexes. For males, the prevalence rate increased from 128 per 100,000 population (95% CI: 109.8–150.1) in 1990 to 160.8 per 100,000 population (95% CI: 139.4–187.5) in 2021, with an EAPC of 0.76 (95% CI: 0.73–0.79, *P* < 0.001). Among females, prevalence rate rose from 286.2 per 100,000 population (95% CI: 247.9–332.5) to 321.7 per 100,000 population (95% CI: 280.0–369.7), with an EAPC of 0.47 (95% CI: 0.42–0.52, *P* < 0.001) (Table 1). From 1990 to 2021, the DALYs rate of RA also showed gender-specific trends. In males, the DALYs rate increased from 27.6 per 100,000 population (95% CI: 20.4–36.3) to 30.6 per 100, 000 population (95% CI: 22.7–40.3), with an EAPC of 0.55 (95% CI: 0.39–0.71, *P* < 0.001). In contrast, among females, the DALYs rate decreased slightly from 57.6 per 100,000 population (95% CI: 44.3–74.0) in 1990 to 54.1 per 100,000 population (95% CI: 39.3–72.6) in 2021, with an EAPC of-0.04 (95% CI:-0.15–0.07, *P* = 0.442) (Table 1).

**Figure 1:**
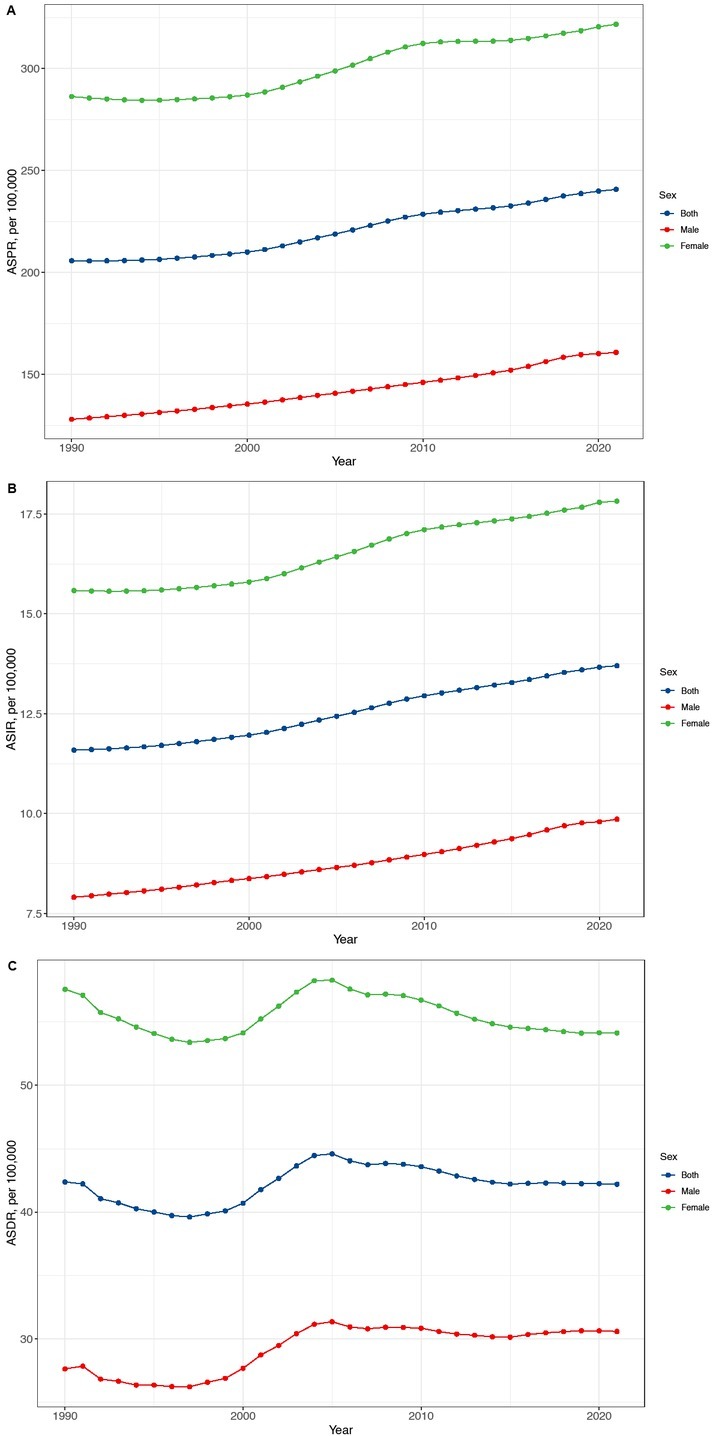
Temporal trend of RA ASPR (A), ASIR (B) and ASDR (C) in China from 1990 to 2021 for men, women, and both sexes. RA: rheumatoid arthritis, ASPI:age-standardized prevalence rate, ASIR:age-standardized incidence rate, ASDR: age-standardized DALYs rate.

In 1990, RA burden in females was notably higher, with incident cases, prevalent cases, and DALYs totaling 85, 500 (95% CI: 74,200–97,400), 1, 413, 100 (95% CI: 1,210, 800–1,646,800), and 277,200 (95% CI: 209,700–360,600), respectively, comprising 66.9%, 69.2%, and 68.8% of the national totals (Table 1). By 2021, these numbers in females had increased to 156, 700 incident cases (95% CI: 137,300–179,800), 3, 196, 700 prevalent cases (95% CI: 2,798,200–3,652,400), and 543, 500 DALYs (95% CI: 400,100–721,500), representing 63.4%, 67.2%, and 65.2% of the national totals, respectively. The burden of RA remained consistently higher among females than males throughout this period (Figure 1).

### National Trends by Age Group

Nationwide, the largest increase in the incidence rate of RA from 1990 to 2021 occurred in the 15–19 year age group, which rose from 5.6 per 100, 000 population (95% CI: 3.1–8.8) in 1990 to 7.6 per 100, 000 population (95% CI: 4.4–11.7) in 2021, with an EAPC of 1.05 (95% CI: 1.01–1.08, *P* < 0.001). This was followed by the 70–74 year age group, which increased from 23.8 per 100, 000 (95% CI: 13.9–37.9) in 1990 to 32.4 per 100, 000 (95% CI: 19.6–49.4) in 2021, with an EAPC of 1.04 (95% CI: 1.01–1.07, *P* < 0.001). Subsequent age groups with notable increases included the 65–69 year (EAPC 0.97 [95% CI: 0.93–1.01], *P* < 0.001), 75–79 year (EAPC 0.94 [95% CI: 0.86–1.01], *P* < 0.001), 20–24 year (EAPC 0.87 [95% CI: 0.83–0.91], *P* < 0.001) , and 80–84 year age groups (EAPC 0.86 [95% CI: 0.75–0.97], *P* < 0.001) (Table 2).

**Table 2:** Number and age-standardized rate of RA prevalence, incidence, and DALYs in China in 1990 and 2021 for different age groups

	1990		2021		EAPC in rate, 1990-2021	*P* value
	Cases (*103)	Rate	Cases (*103)	Rate		
Incidence (95% CI)						
15–19	7.1 (4, 11.1)	5.6 (3.1, 8.8)	5.7 (3.3, 8.7)	7.6 (4.4, 11.7)	1.05 (1.01, 1.08)	<0.001
20–24	11 (7.2, 15.4)	8.3 (5.5, 11.7)	7.8 (5.3, 10.8)	10.7 (7.2, 14.7)	0.87 (0.83, 0.91)	<0.001
25–29	12.1 (6.9, 18.9)	11 (6.3, 17.2)	11.1 (6.7, 17.0)	12.8 (7.7, 19.7)	0.62 (0.57, 0.68)	<0.001
30–34	12.1 (7.5, 17.2)	13.7 (8.5, 19.5)	18.6 (11.6, 26.2)	15.4 (9.6, 21.6)	0.47 (0.42, 0.52)	<0.001
35–39	15.5 (8.7, 23.3)	16.9 (9.5, 25.5)	19.5 (11.2, 28.5)	18.4 (10.6, 26.9)	0.40 (0.35, 0.46)	<0.001
40–44	12.7 (7.3, 18.8)	18.9 (10.9, 28.1)	18.8 (11.2, 27.0)	20.6 (12.2, 29.5)	0.39 (0.34, 0.45)	<0.001
45–49	10.3 (6.3, 15.6)	20 (12.2, 30.3)	24.1 (14.9, 36.2)	21.8 (13.5, 32.8)	0.37 (0.31, 0.44)	<0.001
50–54	10.1 (6.3, 14.9)	21.2 (13.2, 31.2)	28.3 (17.8, 40.9)	23.4 (14.7, 33.9)	0.38 (0.33, 0.44)	<0.001
55–59	9.9 (6.0, 15.5)	22.9 (13.8, 35.8)	28.4 (17.6, 43.2)	25.8 (16.0, 39.3)	0.41 (0.38, 0.45)	<0.001
60–64	8.3 (4.6, 13.2)	23.5 (13.1, 37.3)	20.6 (12.2, 31.3)	28.2 (16.7, 42.9)	0.63 (0.59, 0.67)	<0.001
65–69	6.3 (3.4, 9.6)	23.2 (12.5, 35.3)	23.4 (13.9, 34.1)	30.5 (18.1, 44.5)	0.97 (0.93, 1.01)	<0.001
70–74	4.5 (2.6, 7.1)	23.8 (13.9, 37.9)	17.3 (10.4, 26.3)	32.4 (19.6, 49.4)	1.04 (1.01, 1.07)	<0.001
75–79	2.9 (1.9, 3.9)	25.1 (17.0, 34.6)	11.4 (8.1, 15.0)	34.4 (24.4, 45.4)	0.94 (0.86, 1.01)	<0.001
80–84	1.2 (0.7, 1.7)	22.3 (13.6, 32.3)	6 (3.8, 8.5)	30.1 (19.0, 42.8)	0.86 (0.75, 0.97)	<0.001
85+	0.3 (0.2, 0.4)	14.3 (8.6, 20.8)	2.3 (1.4, 3.3)	17.5 (11.0, 25.1)	0.55 (0.43, 0.68)	<0.001
Prevalence (95% CI)						
15–19	36.4 (22.7, 52.7)	28.8 (17.9, 41.6)	29 (18.8, 41.7)	38.8 (25.1, 55.9)	1.02 (0.98, 1.05)	<0.001
20–24	80 (51.3, 115.1)	60.6 (38.8, 87.2)	59.5 (39.4, 84.1)	81.2 (53.9, 115.0)	1.02 (0.97, 1.06)	<0.001
25–29	113.4 (78.0, 154.6)	103.2 (71.0, 140.7)	114.7 (80.5, 154.5)	132.7 (93.1, 178.7)	0.92 (0.87, 0.97)	<0.001
30–34	136.6 (99.8, 190.3)	154.8 (113.1, 215.7)	232.8 (172.6, 313.2)	192.1 (142.5, 258.5)	0.79 (0.75, 0.84)	<0.001
35–39	200.1 (148.7, 264.4)	219 (162.8, 289.5)	277.7 (209.8, 360.8)	262 (198.0, 340.5)	0.70 (0.65, 0.74)	<0.001
40–44	195.4 (144.0, 248.0)	291.2 (214.6, 369.6)	311.8 (238.0, 390.6)	340.6 (260.1, 426.7)	0.62 (0.57, 0.68)	<0.001
45–49	189.3 (144.2, 238.2)	366.7 (279.3, 461.4)	464.2 (360.4, 577.1)	420.8 (326.7, 523.1)	0.54 (0.49, 0.60)	<0.001
50–54	210.6 (167.5, 257)	441.5 (351.2, 538.7)	602.1 (481.4, 727.4)	498.2 (398.4, 601.8)	0.48 (0.43, 0.52)	<0.001
55–59	224.6 (186.1, 270.3)	517.9 (429.0, 623.2)	639.9 (534.3, 763.8)	582 (486.0, 694.7)	0.43 (0.39, 0.47)	<0.001
60–64	210.1 (176.6, 251.3)	594.6 (499.8, 711.1)	482.4 (408.5, 571.1)	660.7 (559.6, 782.3)	0.41 (0.37, 0.45)	<0.001
65–69	179.3 (150.9, 213.8)	657.1 (553.2, 783.8)	565.5 (483.0, 667.4)	737.3 (629.7, 870.1)	0.46 (0.42, 0.50)	<0.001
70–74	127.3 (107.2, 150.7)	676.2 (569.9, 800.7)	415.3 (351.4, 487.2)	779.2 (659.4, 914.1)	0.54 (0.50, 0.59)	<0.001
75–79	78.4 (67.0, 91.7)	688.8 (588.8, 805.5)	273.9 (235.7, 317.2)	826.9 (711.6, 957.7)	0.63 (0.60, 0.65)	<0.001
80–84	36.7 (31.8, 42.3)	692.1 (600.5, 798.5)	169.8 (148.9, 194.0)	857.7 (752.1, 980.3)	0.72 (0.71, 0.73)	<0.001
85+	13.1 (11.2, 15.3)	645.2 (550.8, 751.2)	105.2 (91.8, 121.5)	803.5 (700.7, 927.9)	0.73 (0.69, 0.77)	<0.001
DALYs (95% CI)						
15–19	8.3 (5.8, 11.8)	6.6 (4.6, 9.3)	4.9 (3.0, 7.5)	6.5 (4.0, 10.0)	0.12 (0.03, 0.21)	0.012
20–24	15.2 (9.9, 22.4)	11.5 (7.5, 17.0)	9.6 (5.7, 14.8)	13.1 (7.8, 20.2)	0.55 (0.46, 0.65)	<0.001
25–29	19.3 (12.6, 28.6)	17.5 (11.5, 26.1)	17.4 (10.8, 26.9)	20.1 (12.5, 31.1)	0.63 (0.52, 0.73)	<0.001
30–34	23.2 (15.3, 34.6)	26.3 (17.4, 39.2)	35 (22.7, 54.0)	28.9 (18.7, 44.6)	0.48 (0.38, 0.58)	<0.001
35–39	34.1 (23.2, 49.2)	37.4 (25.4, 53.8)	41.7 (27.8, 61.2)	39.4 (26.2, 57.7)	0.34 (0.25, 0.43)	<0.001
40–44	33.8 (23.2, 48.1)	50.4 (34.6, 71.7)	47.3 (30.3, 69.2)	51.7 (33.1, 75.6)	0.27 (0.16, 0.37)	<0.001
45–49	33.1 (23.4, 46.6)	64.1 (45.3, 90.2)	71.1 (47.5, 102.2)	64.5 (43.0, 92.6)	0.21 (0.11, 0.31)	<0.001
50–54	39.1 (28.4, 53.0)	81.9 (59.5, 111.0)	95.4 (66.4, 133.2)	78.9 (54.9, 110.2)	0.02 (-0.06, 0.11)	0.569
55–59	43.8 (33.3, 57.1)	101.1 (76.7, 131.6)	105.7 (77.1, 142.0)	96.1 (70.1, 129.2)	-0.08 (-0.17, 0.02)	0.113
60–64	42.7 (33.2, 55.7)	121 (94.0, 157.7)	84.1 (62.7, 111.7)	115.2 (85.8, 153.0)	0.00 (-0.13, 0.13)	0.975
65–69	38.9 (30.5, 50.1)	142.5 (112.0, 183.8)	102.8 (77.9, 132.6)	134 (101.5, 172.9)	-0.02 (-0.16, 0.12)	0.776
70–74	30.8 (24.6, 38.7)	163.7 (130.8, 205.8)	83.3 (64.7, 105.7)	156.3 (121.5, 198.2)	0.08 (-0.08, 0.24)	0.326
75–79	21.1 (17.2, 25.7)	185.4 (150.9, 225.4)	59.5 (46.9, 74.2)	179.6 (141.5, 224.0)	0.09 (-0.08, 0.26)	0.278
80–84	10.2 (8.4, 12.5)	192.2 (158.0, 235.4)	40.8 (31.7, 49.9)	206.3 (159.9, 252.0)	0.47 (0.30, 0.64)	<0.001
85+	4.6 (3.9, 5.7)	227.1 (189.8, 278.6)	32.8 (25.6, 39.6)	250.3 (195.5, 302.0)	0.63 (0.31, 0.96)	<0.001

RA: rheumatoid arthritis, DALYs: disability-adjusted life years, EAPC: estimated annual percentage change, CI: confidence interval.

For prevalence rates from 1990 to 2021, the 15–19 and 20–24 year age groups exhibited the largest increases. The prevalence rate in the 15–19 year age group rose from 28.8 per 100, 000 (95% CI: 17.9–41.6) to 38.8 per 100, 000 (95% CI: 25.1–55.9) with an EAPC of 1.02 (95% CI: 0.98–1.05, *P* < 0.001). In the 20–24 year age group, it increased from 60.6 per 100, 000 (95% CI: 38.8–87.2) to 81.2 per 100, 000 (95% CI: 53.9–115.0), with an EAPC of 1.02 (95% CI: 0.97–1.06, *P* < 0.001). Additional age groups with substantial increases included the 25–29 year (EAPC 0.92 [95% CI: 0.87–0.97], *P* < 0.001) , 30–34 year (EAPC 0.79 [95% CI: 0.75–0.84], *P* < 0.001), 85+ year (EAPC 0.73 [95% CI: 0.69–0.77], *P* < 0.001), 80–84 year (EAPC 0.72 [95% CI: 0.71–0.73], *P* < 0.001) , and 35–39 year age groups (EAPC 0.70 [95% CI: 0.65–0.74], *P* < 0.001) (Table 2).

For the DALYs rate, the greatest increases from 1990 to 2021 were observed in the 25–29 and 85+ year age groups. The 25–29 year group rose from 17.5 per 100, 000 (95% CI: 11.5–26.1) in 1990 to 20.1 per 100, 000 (95% CI: 12.5–31.1) in 2021, with an EAPC of 0.63 (95% CI: 0.52–0.73, *P* < 0.001), while the 85+ year group increased from 227.1 per 100, 000 (95% CI: 189.8–278.6) to 250.3 per 100, 000 (95% CI: 195.5–302.0), with an EAPC of 0.63 (95% CI: 0.31–0.96, *P* < 0.001). Additional age groups with significant increases included the 20–24 year (EAPC 0.55 [95% CI: 0.46–0.65], *P* < 0.001), 30–34 year (EAPC 0.48 [95% CI: 0.38–0.58], *P* < 0.001), 80–84 year (EAPC 0.47 [95% CI: 0.30–0.64], *P* < 0.001) , 35–39 year (EAPC 0.34 [95% CI: 0.25–0.43], *P* < 0.001), and 40–44 year (EAPC 0.27 [95% CI: 0.16–0.37], *P* < 0.001) age groups (Table 2).

In 2021, incident RA cases for each subgroup among population aged 15–44 were 5, 700 (15–19 years), 7, 800 (20–24 years), 11, 100 (25–29 years), 18, 600 (30–34 years), 19, 500 (35–39 years), and 18, 800 (40–44 years), contributing to 2.3%, 3.2%, 4.5%, 7.5%, 7.9%, and 7.6% of the total, respectively, and collectively accounting for 33% of all incident cases. Among the middle-aged 45–59 group, incident cases for each subgroup were 24, 100 (45–49 years), 28, 300 (50–54 years), and 28, 400 (55–59 years), representing 9.8%, 11.4%, and 11.5% of the total, respectively, summing to 32.7% of total incident cases. Among the elderly aged 60–85+, incident cases for each subgroup were 20, 600 (60–64 years), 23, 400 (65–69 years), 17, 300 (70–74 years), 11, 400 (75–79 years), 6, 000 (80–84 years), and 2, 300 (85+ years), corresponding to 8.3%, 9.5%, 7.0%, 4.6%, 2.4%, and 0.9% of the total, respectively, also summing to 32.7% (Table 2). Thus, the RA incidence rates across young (15–44), middle-aged (45–59), and elderly (60–85+) populations in 2021 were approximately balanced at a 1∶1∶1 ratio.

**Figure 2:**
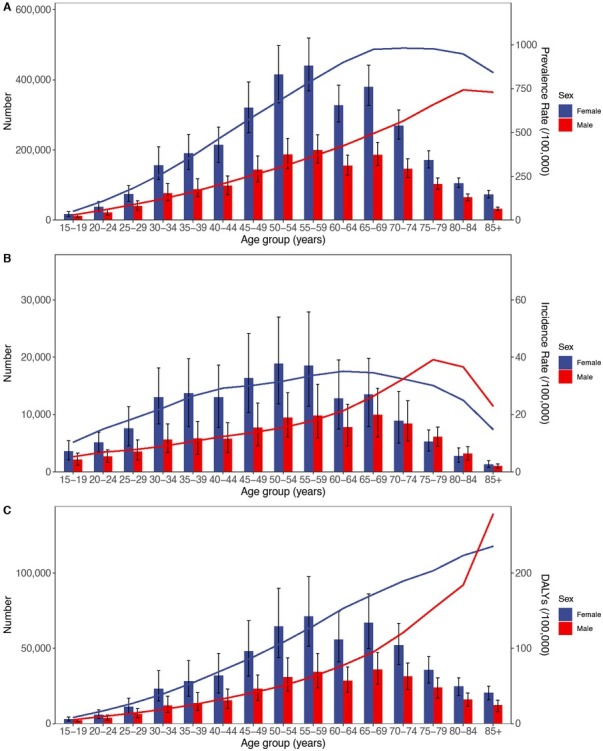
Age-specific numbers and rates of prevalence, incidence, and DALYs of RA by age and gender in 2021. (A) Prevalence, (B) Incidence, and (C) DALYs. DALYs, Disability adjusted life years; RA, Rheumatoid arthritis.

In 2021, the numbers of RA incident cases, prevalent cases, and DALYs in both males and females increased with age, peaking in the 55–59 age group. The total numbers among females were 1.5–2.5 times those of males. A significant decrease was observed in the 60–64 age group, followed by an increase in the 65–69 group. After 65, the numbers of incident cases, prevalent cases, and DALYs generally declined with advancing age (Figure 2).

### Prediction of the RA Prevalence

**Figure 3:**
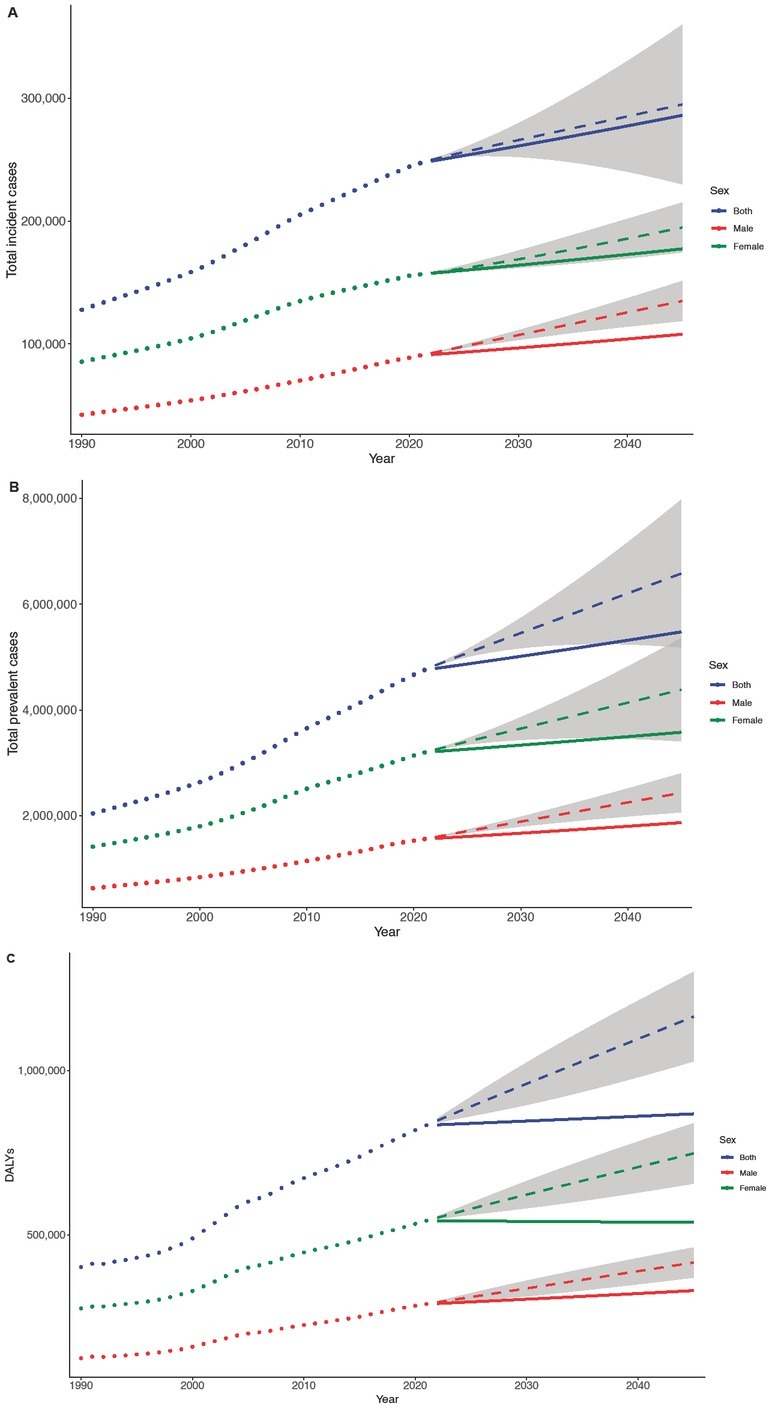
BAPC projections of RA burden by sex in China. Trends for total incident cases. (A), total prevalent cases (B), and total DALYs (C). The dotted line refers to the observed value (before 2021) and the solid line refers to the projected value (after 2021). The shading indicates whether the rate remained stable (baseline reference), decreased by 1% per year (lower limit), or increased by 1% per year (upper limit). DALYs, Disability adjusted life years; BAPC, Bayesian age-period-cohort.

According to the prediction, it is expected that by 2045, the total number of incident RA cases in China will reach 286, 169 (95% CI: 230,133–359,857). Among these, there will be 107,924 male cases (95% CI: 88,717–151,172) and 177,437 female cases (95% CI: 174,656–215,035). The total numbers of prevalent cases and DALYs are projected to reach 5,476, 591 (95% CI: 5,182,249–7,976,695) and 868,511 (95% CI: 728,371–1,099,729), respectively (Figure 3). Compared to 2021, these figures represent increases of 15.7%, 13.2%, and 4.2% in incident cases, prevalent cases, and DALYs, respectively, suggesting a steady rise in RA burden in China over the coming decades.

## Discussion

To our knowledge, this is the first study to estimate the prevalence of RA in China from 1990 to 2021. Over the past 32 years, both the incidence and prevalence rates of RA in China had risen significantly, while the DALYs rate had increased slightly. By 2021, China recorded 247, 300 incident cases (95% CI: 216,200–283,000), 4, 755, 500 prevalent cases (95% CI: 4,141,200–5,452,500), and 833,800 DALYs (95% CI: 621,500–1,083,500). Compared with 1990, incident cases, prevalent cases, and DALYs in 2021 were approximately 1.9, 2.3, and 2.1 times higher, respectively.

In 2021, RA prevalence in China was 4,755, 500 cases, representing 0.34% of the total population (1,412, 600,000). This finding aligns with regional and global estimates. Cross *et al*. reported a global RA prevalence of 0.24% (95% CI: 0.23%-0.25%) from 1990 to 2010.^[3]^ Similarly, Rudan *et al*. found that Southeast Asia’s RA prevalence was 0.40% (95% CI: 0.23%–0.57%) during 2000–2010.^[4]^ In a more recent study, Finckh *et al*. estimated RA prevalence across different regions based on GBD 2017 data, with values of 0.32% in South Asia, 0.21% in Central Asia, 0.19% in East Asia, and 0.10% in Southeast Asia.^[15]^ Almutairi *et al*. further estimated a global average point prevalence of RA from 1980 to 2019 at 0.56% ([standard deviation, SD] = 0.51) and an Asian average of 0.34%, while the period prevalence averaged 0.51% globally and 0.34% in Asia.^[7]^ Differences in RA prevalence across studies may be attributed to variations in prevalence metrics (point prevalence *vs*. period prevalence), differences in study periods and regions, and data quality and sources. The findings of this study align closely with those of Almutairi *et al*., suggesting that the RA prevalence in China approximates the average level observed across Asia.

This study’s findings indicated that from 1990 to 2021, theburden of RA in females was significantly higher than in males. This observation aligns with previous studies by Rudan *et al*.,^[4]^ Li *et al*., Hitchon *et al*.,^[5]^ and Julieth *et al*.,^[6]^ Rudan *et al*., reported that in 2000, there were 2.6 million (95% CI: 1.85–3.34) male RA patients and 12.21 million (95% CI: 9.78–14.67) female RA patients in low- and middle-income countries (LMICs); by 2010, the number of male patients rose to 3.16 million (95% CI: 2.25–4.05) and female patients to 14.87 million (95% CI: 11.91–17.86).^[4]^ Li *et al*. reported that, globally, young populations in 2019 experienced incidence rates of 3.26 per 100, 000 (95% CI: 2.45–4.32) for males and 7.66 per 100, 000 (95% CI: 5.82–10.00) for females.^[8]^ Hitchon *et al*. observed that RA prevalence in Manitoba, Canada, was 1.0% for females compared to 0.53% for males in 2010.^[5]^ Julieth *et al*. noted that by 2019, Colombia had recorded a female-to-male RA ratio of 5.2∶1, with a total of 81, 386 patients.^[6]^ These studies consistently highlighted a gender disparity in RA burden, with females experiencing a notably higher impact than males. Potential explanations for this gender disparity include hormonal influences. Androgens are known for their anti-inflammatory effects, while estrogens exert a dual role on the immune system, downregulating inflammatory pathways while upregulating immunoglobulin production. Decreases in estrogen levels may elevate risk, while prolonged estrogen exposure seems to offer some protective effects. This dynamic may partially account for the higher risk of RA observed among middle-aged and older females.^[16, 17, 18, 19, 20, 21, 22, 23, 24]^ Therefore, addressing the gender disparity in RA by developing gender-specific treatment approaches should be prioritized in future RA research and management strategies.

From an age-based perspective, RA incidence in 2021 was approximately equal across three age groups: 15–44, 45–59, and 60–85+. The relatively high RA burden among younger populations (15–44 years) Li *et al*. reported a global increase in RA incidence and prevalence rates among adolescents and young adults (AYAs) aged 10–24 years from 1990 to 2019.^[8]^ Both studies underscore the importance of addressing RA burden among younger populations, a demographic often underrepresented in RA research.

Although the exact etiology is not yet clear, increasing evidence suggests that the onset and development of RA is a multi-factorial interaction, closely related to genetic and environmental factors. According to previous studies, genetic influences account for 50%–60% of the risk of developing rheumatoid arthritis,^[25,26]^ while the remainder may be explained by environmental effects. A well-established link exists between smoking and RA,^[27, 28, 29, 30, 31]^ and an increasing body of evidence suggests a correlation between environmental pollutants and RA onset. Three epidemiological studies from the United States, Canada, and Sweden have associated air pollution with RA pathogenesis.^[32]^ Alsaber *et al*. investigated the correlation between air pollutants and RA activity through regression models and found that nitrates and sulfur dioxide were significant risk factors for the development of RA.^[33]^ Adami *et al*. assessed the potential association between air pollutants and RA in the Verona region, and a study among 888 RA patients showed that air pollution was related to high levels of C-reactive protein (CRP), the severity of RA disease, and its recurrence due to poor response to biologic therapy.^[34]^ Lei *et al*. further identified that exposure to volatile organic compounds (VOCs) was linked with RA onset.^[35]^ To reduce RA risk, prevention efforts should emphasize controlling modifiable factors, including smoking cessation and minimizing exposure to environmental pollutants. Addressing these factors could lower RA risk, particularly through lifestyle modifications and occupational safety measures to reduce exposure. This study has several limitations. First, the GBD raw data were collected from various databases with differing quality, potentially contributing to considerable heterogeneity in the results. Second, the data obtained from the GBD are modeled estimates rather than original observational data. While advancements in data processing and modeling techniques, such as DisMod-MR 2.1, enhance the accuracy of GBD estimates, significant improvements would require more robust and comprehensive primary data collection. Third, due to a lack of data from various provinces and regions, this study was unable to conduct a regional analysis of RA burden within China. Additionally, the GBD database was only updated to 2021, so recent data from the past two years were not included.

Rheumatoid arthritis is a relatively common and harmful autoimmune disease, representing a substantial portion of the global disease burden. This study leveraged GBD 2021 data to analyze RA epidemiological trends in mainland China from 1990 to 2021. Over the past 32 years, both the incidence and prevalence rates of RA in China have shown substantial growth and are anticipated to continue a stable upward trend in the coming decades. In recent decades, with the introduction of early treatment and Treat-to-Target (T2T) strategies, RA outcomes, including reductions in disease severity, disability, and mortality, have significantly improved in developed countries.^[9]^ In China, however, patient awareness of these strategies to limit disease activity, radiographic progression, and functional impairment appears suboptimal. Additional barriers, such as high treatment costs, further limit the adoption of these management recommendations.^[10,11]^ Consequently, there had been a slight increase in RA-related DALYs.

To address the rising RA burden in China, efforts should prioritize national awareness campaigns emphasizing the importance of early prevention, timely diagnosis, and effective treatment. Besides, focused initiatives targeting younger and female populations are also very crucial for improving RA outcomes and reducing the associated disease burden.
